# Automated Tracking Systems for the Assessment of Farmed Poultry

**DOI:** 10.3390/ani12030232

**Published:** 2022-01-19

**Authors:** Suresh Neethirajan

**Affiliations:** Farmworx, Adaptation Physiology Group, Department of Animal Sciences, Wageningen University & Research, 6700 AH Wageningen, The Netherlands; suresh.neethirajan@wur.nl

**Keywords:** poultry behaviour, target tracking, deep learning, precision livestock farming, poultry production systems

## Abstract

**Simple Summary:**

With the advent of artificial intelligence, the poultry sector is gearing up to adopt and embrace sensor technologies to enhance the production and the welfare of birds. Automated tracking and tracing of poultry birds has several advantages in poultry farms: overcoming the subjectivity of human measurements, enhancing the ability to provide quality care for the birds during their life on the farm, providing the ability to predict events and thereby enabling timely interventions, and many more. However, the technologies behind automated tracking systems are not ripe due to the lags in algorithms and practical implementation issues. This mini review provides a brief critical assessment of the current and recent advancements of automated tracking systems in the poultry industry and offers an outlook on future directions.

**Abstract:**

The world’s growing population is highly dependent on animal agriculture. Animal products provide nutrient-packed meals that help to sustain individuals of all ages in communities across the globe. As the human demand for animal proteins grows, the agricultural industry must continue to advance its efficiency and quality of production. One of the most commonly farmed livestock is poultry and their significance is felt on a global scale. Current poultry farming practices result in the premature death and rejection of billions of chickens on an annual basis before they are processed for meat. This loss of life is concerning regarding animal welfare, agricultural efficiency, and economic impacts. The best way to prevent these losses is through the individualistic and/or group level assessment of animals on a continuous basis. On large-scale farms, such attention to detail was generally considered to be inaccurate and inefficient, but with the integration of artificial intelligence (AI)-assisted technology individualised, and per-herd assessments of livestock became possible and accurate. Various studies have shown that cameras linked with specialised systems of AI can properly analyse flocks for health concerns, thus improving the survival rate and product quality of farmed poultry. Building on recent advancements, this review explores the aspects of AI in the detection, counting, and tracking of poultry in commercial and research-based applications.

## 1. Introduction

Today’s demands for increased livestock production result in various challenges for the animals they pertain to. A balance is needed between the quantity and quality of poultry production. However, farmers must worry about maximizing profits, a need that has promoted a prioritization of production over aspects such as welfare. Flock size and growth are commonly maximised in minimal spaces to offset low margins for farmers. Societal pressures towards sustainability also influence minimal inputs for poultry farming aspects such as land, labour, and natural resource usage. These efforts may lead to increased poultry production with decreased production time and resource usage, but they have also unintentionally led to the proliferation of harmful genetic alterations and the increase in associated diseases. The solution to these complex agricultural needs is to assist farmers with automated surveillance of the animals. Through the continuous and automated monitoring of animals, farmers are able to detect welfare and production concerns in a manner that is both quick and reliable. With the integration of modern technological advances, poultry farming has the opportunity to grow in terms of production quantity and animal care quality with minimal added expense.

On a global scale, 69 billion chickens are raised for meat production every year [1], but not all of them make it to people’s plates. In the UK alone, over a 3-year period between 2016 and 2019, about 61 million chickens were rejected for human consumption due to defects and diseases in slaughterhouses [2]. The threshold set by the Spanish agency for Food Safety and Nutrition on the rejection of chickens before processing in slaughterhouses, is 2% per annum [3]. In a Turkish study [4], it was estimated that approximately 0.4% of broiler chickens are dead-on-arrival before the process of slaughtering under commercial conditions. Globally, several million chickens do not survive the rearing process, and are possibly rejected at the slaughterhouse because of illnesses, scratches, bruises, and other signs of welfare failures. Considering the difference between food accessibility and hunger for some people, and for farmers, rejection of chickens at slaughterhouses can be a great source of profit loss. This statistic also makes a huge difference for the animals, as it suggests that millions of chickens bred for meat suffer from unmanaged, painful, and possibly deadly medical (pathological) conditions each year. With better diagnostics and agricultural management, fewer resources would be wasted, more chickens could be produced, and less suffering would be faced by these animals.

There is a need to increase agricultural capabilities to detect anomalies in chicken behaviour and health, and thereby welfare, without increasing a need for manual labour, and for that, automated systems are needed. Automated systems have been studied and proven to be capable of accurately collecting data related to the following needs:

- Individual tracking, even in large groups of animals that are condensed in a confined space.
- Phenotype assessment and analysis for the non-invasive understanding of genotypes, which are important for resilient breeding methods.
- Identification of the needs of individual animals in relation to welfare.
- Continuous data-collecting capabilities that cannot be replicated by humans.
- Assessment of activity and changes on a flock level.
- Early direction of behavioural and physical shifts in comparison to past flocks.
- Analysis of nuances related to welfare-focused farming, such as the preferences in light intensity for individuals or groups of agricultural birds.
- Long-range use for the non-disruptive observation of fearful and free-range livestock.
- Bone fracture assessment for immediate intervention.

## 2. Need for Automated Poultry Surveillance

Poultry and eggs are a major source of dietary protein for people across the globe [5]. As a result, these animal food sources must be produced in a way that minimises their cost and maximises their availability if poultry is going to remain a major food source as the human population continues to grow. The profitability and productivity of commercial poultry farming depend on regular monitoring of the birds, and minimal human labour to maintain its affordability [6]. Recently, in June 2021, the European Commission set a goal to phase out the use of cages for farmed animals by the year 2027. This created a need for redesigning the poultry housing systems using an enhanced understanding of the range of behaviour and locomotion of laying hens and broilers. The modern solution to this issue can be found in technological advances that are both growing in accuracy and decreasing in price. Introducing artificial intelligence (AI) in poultry farming and management has the potential to improve multiple aspects of the industry. With the ability to accumulate data that triggers informed actions, this technology has the potential to improve animal welfare, minimise the spread of disease, improve breeding standards, and reduce waste [7]. With so many promising implications, it should be no surprise that automated poultry surveillance is receiving plenty of attention in the realm of research.

Many of the production economics of poultry farms depend upon visually accessible aspects, such as the size, weight, and appearance of poultry eggs and meat. This is precisely why computerised, video-based systems are becoming a popular real-time automated tool for poultry processing. It is praised as a non-intrusive and non-invasive option for flock assessment that seriously reduces, and even eliminates, events of unnecessary stress, which are commonly caused by human observation. This aspect makes it a beneficial tool for presenting a wide range of data on animals within a flock and for the sorting and grading of poultry-related products [8].

The detection and prediction of abnormal behaviour and poultry diseases can be accurately managed using automated tracking platforms [9]. These systems are capable of recording data and analysing poultry farming focuses, including flock density, flock floor distribution, heat stress, feeding and drinking behaviours, optical flow patterns, activity, and the detection and counting of laying hens [10]. 

With the continuous focus on enhancing the welfare of chickens and mounting of new evidence towards chicken cognition and emotions [11,12,13,14], there is a dire need for considering the individual needs of chickens. This demands a change in poultry management from a flock-level perspective to an individual bird’s needs. AI technologies enable the identification of individual broilers [15], or laying hens, among hundreds of birds via videos irrespective of similar sizes, shapes, and colours of the feathers. This unique ability enables automated monitoring systems to offer welfare-centred intervention decisions. This technology also permits the use of robust detection of eggs, which will make the tedious and time-consuming task of floor egg collection easier for farmers [16]. Behavioural issues in group-housed turkeys, such as cannibalism, can be rapidly detected and consequently addressed through deep learning techniques [17]. Some systems are even developed to find the location of chickens on a farm for simplified assessment and treatment by farmers [18]. 

## 3. Artificial Intelligence in Poultry Monitoring

Currently, the role of AI in various aspects of society is becoming increasingly obvious to the public, so it is no surprise that this method of management is making its way into food production systems. Computerised monitoring technology promises to fulfil the growing criteria for improved poultry production management, including conversion of the feed ratios and profitability [19].

### 3.1. Computer Vision Technology

Welfare factors related to farm management can be better understood by monitoring poultries’ natural processes and responses. Computer video systems can assess and determine a wide range of data at a time, including housing management, weight measurement, behaviour, detection of diseases, slaughtering processes, egg quality, and carcass quality checking [6,19]. 

### 3.2. Components of Machine Vision for Poultry Tracking

A computer tracking and monitoring technology for various poultry processes consists of two main parts [19]. The first of which is the hardware. Hardware is recognised as the physical components of these systems, which include computing instrumentation, data-acquisition hardware, lighting, wiring, and other tangible components. Advancements in hardware are the primary reason for the development of vision technology in poultry farming. There are three key components of functional hardware in computer vision systems:

- Cameras and various lenses suited to the environment and assigned task.
- Lighting units.
- Mounts that allow for the full view of an observed farming space, without interrupting normal poultry functions.

The second component is software. This includes the programs and other operating information needed for the hardware to perform its specified function. Software is specially designed for data acquisition and data analysis, especially in the field of agriculture, where it must be altered to suit the species of interest. A data-acquisition software system performs its role in the storage and selection of good quality images (or videos) that are produced by the cameras. Data-analysis platforms help in the processing of images using algorithms suited to the data and research needs [19]. 

### 3.3. Types

Computerised, visual-analysis systems exist in two major forms, which are identified as machine learning-based systems and deep learning-based systems.

#### 3.3.1. Machine Learning-Based Systems

Machine learning-based computer vision systems follow specific image analysis protocols and a specially designed algorithm. The basic workflow of a machine learning-based system for poultry monitoring is as follows [6]:

- Acquisition of image: focused on depth or RGB images.
- Pre-processing of image: normalization, resizing, and colour-space transformation.
- Region of interest (ROI) segmentation: background removal or subtraction, ellipse modelling, and other focus-enhancing alterations.
- Features extraction: optical flow meter, locomotor, and morphological features.
- Modelling: machine learning-based algorithms.
- Regression: monitoring of bioprocesses and bioresponses.

#### 3.3.2. Deep Learning-Based Systems

Deep learning-based computer vision systems are a recent advancement in automatic livestock observation, which simplify associated data processing. Various processes of machine learning systems, such as segmentation, feature extraction, and selection, are time consuming and subjective laborious tasks. It is also important to note that the performance of these algorithms must change in relation to sensor sensitivity [6]. The most important feature of a deep learning system is its ability to directly process the image, thus eliminating older laborious processes by using a deep neural network (DNN). These deep learning models generally provide higher accuracy than machine learning, making them better suited to the observation of large flocks [6]. Deep learning systems also solve the common complications with multiple object tracking when using a single camera. This is a revolutionary advancement for researchers and farmers since it minimises equipment costs [20].

### 3.4. Applications

Computer vision systems can be adapted to suit a variety of applications, including the following, which are geared towards poultry farming [6]:

- Recognition and identification of images: checking for the presence of poultry in every image.
- Detection of object: locating the exact position of poultry in every image.
- Classification of image: classifying the identified poultry as sick or absent.
- Segmentation: identifying the watering and feeding structures in every image.
- Recognition of specific objects: noting the behaviours exhibited by members of a flock.

## 4. Milestones in the Field of Automated Poultry Tracking

Different techniques and methods have been developed in the poultry industry to ensure improved production rates [21]. Among the poultry species farmed globally, chickens are the most common and are produced in the largest quantities. Recent advancements of automated poultry monitoring tools is shown in Table 1.

### 4.1. Individual Chicken Identification in Crowded, Free-Ranging Spaces

Individual tracking of poultry has the benefit of evaluating abnormal behaviour and predicting diseases for immediate treatment, but this can be a near-impossible task in crowded housing situations [22]. For this purpose, scientists devised a system to individually track a bird by using image thresholding, feature engineering, and morphological transformation [23].

The non-invasive integration of a deep regression network has the capacity to greatly enhance the functionality of poultry farms down to the individual level. Various comparative research studies have proven that TBroiler, a poultry tracking algorithm, shows superior results based on pixel error, failure rate, and overlap ratio when compared to older algorithms [9]. Furthermore, researchers have made the system check the relationship between spatial memory and the ranging behaviour of free-range broiler chickens for more in-depth analysis [24]. This method of visual identification is also effective in locating free-range egg layers with great accuracy, allowing robotic collection methods to function efficiently [25].

### 4.2. Detection of Broiler Movements through Optical Flow Patterns

The technological advancement of poultry farms comes with extraordinary animal welfare and productivity improvements on both the individual and flock scale [26]. These advancements have also made it possible for researchers to conduct life-long monitoring of individual birds or entire flocks as needed [27,28]. 

Gait variation between broiler chickens is a tell-tale sign of abnormalities in physical conformation and developmental complications. Normally a human would be tasked with observing and rating the gait of broiler chickens. However, that task consumes a great amount of time and money despite its inaccuracies from human biases and unintentional influences. Automated machine vision cameras can be specially programmed to detect any variation in gait among individuals in a flock at speeds and consistencies that reach far beyond those of a human. Through optical flow patterns, variations in the gait of slow-moving birds are easily identifiable on an individual level, especially in comparison to fast-moving birds that display a uniform motion of individuals [7]. This form of information collection is crucial for the non-invasive analysis of bone strength, keel health, and bone structure in poultry, since it does not require stressful human interactions.

This form of tracking also opens up the opportunity to include 3D camera technology. These cameras use a depth sensor to identify the lameness of individual broiler chickens through body positioning and inactivity that can only be assessed using depth perception [27]. Another technique for assessing lameness in broiler chickens includes the use of an image analysis algorithm that detects motion variables, such as lateral body oscillation, speed, step length, and step frequency [29]. 

### 4.3. Increasing Poultry Productivity through Time-Series Data Mining

Recent advancements in sensor technologies make it easy for farmers to record and measure animal behaviour patterns and provide timely interventions or decision-making capabilities. However, there still are challenges that must be faced in order to achieve a booming poultry production rate [30]. 

Complications such as heat stress are unavoidable in some areas, and the increases in temperatures can cause natural behavioural changes in poultry. Most commonly, heat stress causes broiler chickens to reduce activity, which can skew typical observation results towards inaccurate rates of lameness. Luckily, researchers have proposed a model for recognizing heat stress by making use of the YOLOv3 algorithm. This algorithm increases the accuracy of lameness assessments in flocks to a satisfactory rate of 83% [31].

### 4.4. Image Analysis of Broiler Chicken Behaviour at Different Feeders 

The feeding patterns of poultry, such as broiler chickens, dictate the type of feeders needed for proper nutrition and behavioural control [25]. In one study, three types of feeders were used. These feeders included automatic systems with a partition grid, tubing systems, and tube systems without a barrier grid. Observations were then recorded using computational image data analysis. The activity index was a variable that was not influenced by the feeder type, but other behavioural variables showed some relation. The conclusion of this study showed that the design of poultry feeders has significant impacts and that feeding behaviours can be accurately monitored through automated image analysis [32,33].

### 4.5. Detection of Poultry Diseases Using Deep Learning Systems and Image Analysis

Diseases in confined poultry farming operations spread quickly, leading to welfare issues for the animals and significant financial losses for farmers [34]. In a majority of situations, diseases are not detectable by manual observation. However, certain observable factors signify a healthy flock, such as the spatial distribution of animals [35].

Flock attributes such as spatial distribution are best detected through visual observations. This leads to the use of digital image analysis and deep learning systems. In one study of a modernised system, an Improved Feature Fusion Single Shot Multibox Detector (IFSSD), along with the single shot multibox detector, was proposed as a method of enhanced algorithmic assessment. The IFSSD can improve and enrich the image quality, making it easy to detect sick broiler chickens amongst large flocks [34]. 

Through the usage of optical flow, one can easily detect visual ailments such as dermatitis and hock burn in poultry [36]. Another milestone in modern poultry farming was installing a system to detect *Campylobacter*-positive birds using cameras and analysing optical flow patterns. The detection of high movement peaks and lower mean optical flow patterns indicate that a flock could be carriers of *Campylobacter*—a major cause of gastrointestinal infections in humans [37].

Zoonotic and highly contagious diseases, such as bird flu, cause economic losses to farmers and threaten human health [38]. Historically, such diseases are not recognised and treated until after they have caused significant damage, but the use of real-time image monitoring provides a simple and effective solution [38]. Healthy and sick broiler chickens can be easily differentiated through posture comparisons, allowing farmers to take immediate outbreak-preventing actions.

### 4.6. Infrared Receiver Assessments of Keel Bone Fractures in Laying Hens

The keel is a crucial physiological structure in chickens that is closely related to locomotion. It can be damaged in a number of ways, leading to lameness and even premature death. To better assess the occurrences of keel bone fractures, researchers created a real-time tool for the assessment of laying hens by using infrared receivers. Here, they attached infrared receivers to the legs of the hens and monitored their behaviour patterns [39]. This minimally invasive method of measuring lameness and keel health is practical and effective for the assessment of small flocks, but it is less practical for use in larger poultry farming operations. A per-animal approach in poultry is practically not possible, unlike in the cattle and swine industries. Passive radio-frequency systems function in a similar method to these infrared receivers and provide clear, individualised points of information on the animals within a flock, but they face the same issue of impracticality on large scales [40]. 

### 4.7. Evaluation of Laying Hens’ Light Preferences 

Researchers developed a visually based light preference test system to detect and count laying hens. Light may appear to be a minor aspect of farming, but it can have a significant impact on the laying quantities and life quality of laying hens. In this study, two algorithms were used, one for the analysis of images and the other for weight. The accuracy of this test system was based on automatic visual observation, which made it possible to detect the laying hens in each light compartment along with their number [41].

An automated system was designed to detect the laying hens present in battery cages by using cameras along with the tracking algorithms and automated video technology for the detection of laying hens occupying multiple nests [10,42]. This technology has also served to assess the range of individual layers for better production management. It works by using radio-frequency technology that tags laying hens individually [43]. This method is practical only in larger livestock or research animals, as poultry are generally farmed in large quantities that would require time and large financial investments in order to individually tag an entire flock.

### 4.8. Deep Learning System Detection of Pecking Activity in Grouped-Housed Turkeys 

When turkeys are raised in artificially confined environments, they become more prone to cannibalistic behaviours that can be distinguished through pecking and movement patterns. These behaviours must be caught early and bred out of a gene pool to avoid catastrophic financial losses and unnecessary animal suffering. In one study [17], two metallic balls were used along with a microphone to record pecking audios that suggest behavioural changes. Video data were also recorded by a camera, which was mounted for a top view of the study subjects. Using the data from these two sources, cannibalistic pecking activity measurement in turkeys was demonstrated with convolutional neural network (CNN) models for a full assessment and intervention related to this destructive behaviour [17].

### 4.9. Tracking and Stocking Density Estimation

The monitoring of individual (per animal) poultry birds is a difficult task, even with the use of video-based monitoring applications. The similar appearances of individuals, occlusions, and other technical complications motivated scientists to develop a robust detection, counting, and tracking method that was capable of assessing multiple animals at once. The primary feature of this observation method is its ability to monitor the animals constantly. It can even collect data at night by using infrared cameras through heat map-based classification and evaluation. This technological advancement allows farmers to measure and estimate stock density and behavioural patterns on an individual basis [44]. Deep and shallow vision technologies could also be implemented in the near future to help assess behavioural pattern changes, such as pecking, over time [45,46,47].

**Table 1:** An overview of current research advancements of automated poultry monitoring tools.

Applications	Used Tools and Platforms	Solved Poultry Problems	References
Counting of individual broilers	Camera, TBroiler	Abnormal behaviour; patterns	[9]
Broiler movement	Camera	Various among individuals	[7]
Productivity in broilers	Camera, sensors	Advance treatments for healthy growth	[30]
Behaviour at different feeders	Camera	Choice of feeder design	[32]
Detection of disease	Camera, Improved Feature Fusion Single Shot Multibox Detector (IFSSD)	Outbreak prevention	[36]
Sick broiler assessment	Camera	Disease management	[38]
Keel bone fracture	Infrared receivers	Timely treatments	[39]
Laying hen light preference	Camera, tracking algorithm	Layer detection in cages	[41]
Pecking in turkeys	Camera, microphone, and metallic balls	Assessment of cannibalism	[17]
Tracking in pigs	Camera, sensors	Individual behaviour	[44]
Poultry movement and range behaviour assessment	AI-based algorithms and cameras (multi-object tracking algorithm and single shot multibox detector algorithm)	Group-level poultry movement	[47]
Turkey behaviour identification	Video analytics, multi-object tracking	Turkey health status and behaviour identification	[48]
Thermal comfort of poultry birds	Camera, computer vision	Unrest index and locomotion	[49]
Laying hen behaviour	Camera, AI algorithms	Cluster and unrest behaviour	[50]
Adult free-range hen behaviour investigation	Camera, sensors, AI algorithms	Range use and fearfulness behaviour	[51]
Stocking density of broilers	AI algorithms, machine vision cameras	Relationship between stocking density and feeding/drinking of broilers	[52,53,54]

## 5. Challenges and Future Research Directions

In this paper, we present insightful discussions on the possibilities of AI and sensor technologies for poultry industries and, more specifically, on the tracking of an individual bird and flocks for real-time decision making based on animal measures. Discussions on how leveraging the sensor technologies and machine vision-based methods can provide data in a transparent, accessible, traceable, and decentralised manner have been included. The convergence of AI, along with blockchain capabilities, can provide an efficient way of tracking the birds through rapid data analytics. Being able to observe variations in the data in a real-time fashion will enable farmers and poultry industry personnel to make on-time, rapid decisions without human interventions via innovative analytics. Integration of multimodal data and the quality of the data are the factors that can enable the AI platforms to improve the accuracies of predictive analytics. 

One of the challenges is the ability to integrate the existing sensing systems in the barn-level poultry industry with automated artificial intelligence-enabled platforms. AI algorithms, through automation, can transform routine data collection from being sensor centric to being based on an individual animal’s needs. Automated poultry tracking systems, which are based on the Internet of Things, can offer a variety of benefits such as real-time monitoring, remote tracking via smartphone or dashboard mobile applications, and alerting in case of critical scenarios of welfare-impairment situations. Storage of all the poultry-based data from the numerous machine vision camera systems and sensor technologies offer poultry management personnel easy management by avoiding subjectivity and inaccuracies in the processes of decision making. Practical challenges during implementation include functional issues, readability for non-technical end users, throughput and latency issues, dynamic data control flow, execution efficiency, and data security. In addition to the technical challenges, along with access to a high-speed internet connection for real-time upscaling and downscaling of multimodal data for medium and small-scale farmers, meticulous validation of the tracking and measurement systems and narrower profit margins continue to remain as bottlenecks in implementation. By overcoming the above-mentioned challenges, the full potential of artificial intelligence-enabled tracking systems can offer a new wave of innovations in the poultry sector. 

## 6. Conclusions

Continuous real-time heterogeneous data streaming from multiplexed sensors in the poultry barn for automated decision-making processes is still a challenge. Due to advancements in the CNN, hardware manufacturers have introduced high throughput internet data transfer capabilities of over 10 tera operations per second. However, currently, most of the automated detection and tracking systems in the poultry industry are essentially passive and cannot control or interface with changing the ventilation systems or feed inlet controls or creating alarms or call-for-actions to veterinarians. Automated tracking of poultry platforms requires the detection, selection, and tracking of activity of the poultry without the birds having any prior stored trajectory (motion planning prediction). This is a bottleneck in the image processing aspects, but AI-based prediction algorithms may be able to overcome this barrier. Further challenges in the practical realisation of the automated systems involve multiplexing of sensor technologies, such as ultrasonic, LIDAR, cameras and real-time data processing hardware in achieving the accuracy, and thereby the prediction, of the poultry movement and behaviour. The use of AI-enabled technology in poultry farming is essential for increasing the production rate and improving welfare-based farming practices. Economic losses and suffering can be effectively prevented through the automated visual detection of diseases. These advancements in individual and flock behavioural evaluations will help the poultry agricultural sector grow in production without the need for major sacrifices along the way.

## Data Availability

Not applicable.
